# Proximal Dissection and Rupture of a Popliteal Cyst: A Case Report

**DOI:** 10.1155/2012/292414

**Published:** 2012-10-10

**Authors:** M. H. Abdelrahman, S. Tubeishat, M. Hammoudeh

**Affiliations:** ^1^Rheumatology Section, Department of Medicine, Hamad General Hospital, P.O. Box 3050, Doha, Qatar; ^2^Radiology Department, Hamad Medical Corporation, P.O. Box 3050, Doha, Qatar

## Abstract

Popliteal cysts are swellings in the popliteal fossa due to enlargement of the gastrocnemius semimembranous bursa. These cysts might burst, and they usually rupture posteriorly and inferiorly with severe pain in the calf. We describe a patient with popliteal cyst that dissected proximally and ruptured in the soft tissue of the thigh.

## 1. Case Report

**Figure 1:**
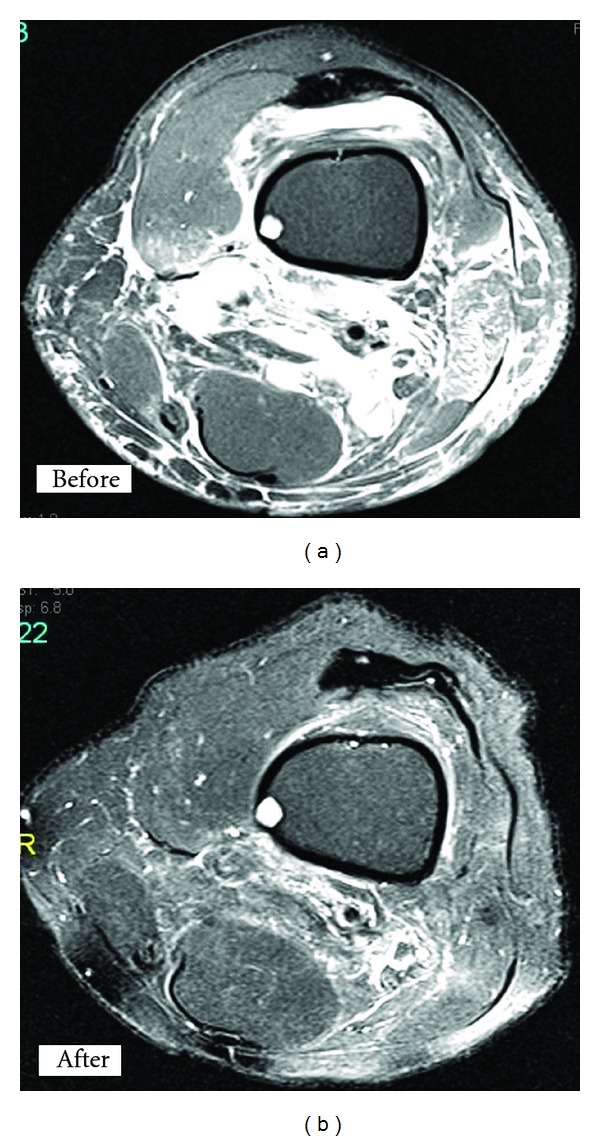
MRI of the Knee showing proximally dissecting ruptured baker cyst before and after treatment.

A 63-year-old male, known to have familial Mediterranean fever (FMF) and maintained on colchicine 0.5 milligram daily, presented with few days history of pain and swelling in the left knee that extended above the knee to the midthigh, with difficulty in bending the knee. He gave no history of fever, trauma, or other joint involvement. Physical examination revealed warm, tender, and markedly swollen left knee with swelling of the distal third of the thigh with 8-centimeter difference in the circumference of both sides above the patella. Knee joint aspiration showed turbid synovial fluid with white blood cells of 19850/microliter, of which neutrophils comprised 94%, lymphocytes 5%, and red blood cells were 850/microliter. Synovial fluid culture did not grow any organism and microscopic examination did not show any crystals. Magnetic resonance imaging (MRI) of the knee showed (Figure 1(a)) multiple cystic collections at the interfacial spaces of the hamstring muscles. The largest lesion measured 7.9 centimeter by 4.6 centimeter, with extensive edematous infiltration involving the soft tissue of the thigh and calf posteriorly, surrounding the above-mentioned cysts, compatible with perforated cyst with a leak of its content into the soft tissue and mild to moderate joint effusion. Patient received intra-articular corticosteroid injection and oral nonsteroidal anti-inflammatory drug, and the pain improved after few days. MRI repeated after two weeks and showed (Figure 1(b)) regression of the cystic collections and resolution of inflammatory changes.

## 2. Discussion

The popliteal or Baker's cyst is a synovial cyst named after Baker in 1877 [1]. These cysts present as swelling in the popliteal fossa due to enlargement of the gastrocnemius semimembranosus bursa, which lies on the medial side of the fossa. They contain synovial fluid, and they usually communicate with the adjacent knee joint space.

Complications of popliteal cysts are dissection, rupture, pseudothrombophlebitis, leg ischemia, nerve entrapment, and compartment syndrome.

These cysts may rupture causing severe pain at the calf, with warmth, erythema, and tenderness. This might be confused with other causes of swelling and pain in the calf like deep vein thrombosis, that is, pseudothrombophlebitis [2]. Differentiation can be made with the help of ultrasound.

Compression syndrome secondary to entrapment of the neurovascular bundle in and around the popliteal fossa is a well-known complication [3]. 

Compartment syndrome, a medical emergency, is another complication of popliteal cyst rupture. Popliteal cyst reported to cause both anterior [4] and posterior [5] compartment syndrome. It requires immediate assessment of compartment pressure and if raised, surgical decompression to prevent permanent deformity [5].

Baker's cyst typically results from the leak of joint fluid through a weakened posteromedial joint capsule into the gastrocnemius semimembranosus bursa, between the medial head of gastrocnemius and the semimembranosus tendons [6]. Popliteal cyst might also dissect away from the popliteal fossa; this is usually in an inferomedial direction, but it can dissect anywhere, for example, anterior [4], intramuscular [7], lateral [8], and proximal [9, 10]. The baker cyst in the case we are presenting is different from other baker cyst cases with proximal dissection. This case ruptured, rather than causing nerve compression [9] or presenting as space occupying lesion [10]. Although, Baker cyst can occur in the setting of inflammatory condition, like rheumatoid arthritis [10]; this case, to the author's knowledge, is the only case to be reported in the background of FMF. We give another example of proximal dissection of a popliteal cyst adding to the two cases in the literature. In conclusion, Baker's cysts can manifest in a wide range of presentations and could easily be missed; doctors should be aware that popliteal cysts could dissect anywhere and do not follow the anatomical planes, therefore, should be considered among the differential diagnosis of masses in the lower limbs.
